# Prostate cancer brain metastases: Monitoring response to treatment with PSMA PET/CT

**DOI:** 10.1016/j.radcr.2024.02.110

**Published:** 2024-03-23

**Authors:** Anas Al-Zubaidi, Samuel Bezold, Peeyush Bhargava, Javier Villanueva-Meyer

**Affiliations:** Department of Radiology, University of Texas Medical Branch, Galveston TX 77555, USA

**Keywords:** Prostate cancer, Brain metastasis, PSMA PET/CT, Radiation therapy

## Abstract

Prostate cancer brain metastases are rare but increasingly recognized with prostate-specific membrane antigen (PSMA) PET/CT. Distinguishing tumor response from postradiation changes are challenging on MRI. PSMA PET/CT may clarify equivocal brain lesions after radiotherapy.

A 71-year-old man with metastatic prostate cancer developed 2 new brain lesions on PSMA PET/CT. Lesions were high PSMA-avid and MRI follow up showed enhancing masses with edema, consistent with metastases. He underwent whole-brain radiation. Follow-up PSMA PET/CT after radiotherapy demonstrated significantly decreased lesion size and activity, with activity lower than blood pool, indicating a treatment response. MRI also showed near-resolution of the lesions.

This case highlights the potential utility of PSMA PET/CT for detecting prostate cancer brain metastases and monitoring treatment response. PSMA PET/CT provides valuable complementary information to MRI for managing irradiated prostate cancer brain metastases.

## Introduction

Prostate cancer is the most common noncutaneous malignancy among men in the United States. Despite being amenable to early detection, there has been an increasing annual incidence of advanced-stage prostate cancer since 2011 [1]. Most prostate cancers are localized at diagnosis, but 4%-25% of patients present with distant metastases [2,3]. Prostate cancer later metastasizes to bone (84%) and less so to lymph nodes (11%), liver (10%), and lung (9%) [4].

Brain metastases remain rare with an incidence of 0.6% to 2.9%. Higher percentages have been observed in autopsy series, suggesting that some brain metastases may remain undetected despite improvement in imaging technique. Most lesions affect the meninges, and about one-third are intra parenchymal. Brain metastases are mostly present in patients with high grade, small cell, neuroendocrine-transformed, and advanced-stage prostate cancer and often associated with advanced widespread metastases elsewhere [5], [6], [7].

Accurate staging and follow-up are critical for optimizing patient management. Multiple imaging modalities play important roles in diagnosis, staging, and surveillance of prostate cancer [8]. Between 2020 and 2023 prostate-specific membrane antigen (PSMA) PET/CT with Ga68 PSMA11 (Locametz, Illucix), F18 DCPF PYL (Pylarify), and F18 Flotufolastat (Posluma) were FDA approved to image prostate cancer patients with suspected recurrence. These agents bind to the PSMA receptors expressed by >90% of prostate cancers, in comparison to the previously approved Fluciclovine (Axumin) PET/CT which is taken up by the upregulated amino acid transporters seen in prostate cancer cells. All these agents have extremely low or absent brain activity, thus lesions seen on PSMA PET/CT are very conspicuous. These agents have been rapidly incorporated clinically for staging and follow up of prostate cancer patients. It is conceivable that with widespread use of PSMA imaging that brain metastases which could have previously gone undetected may be now appreciated.

Benign meningiomas and gliomas may be positive with PSMA, and other cancer metastases may express PSMA avidity. Brain only prostate metastases may be recognized with the recently expanded use of PSMA [9]. Additionally, response to treatment of prostate cancer brain metastases may be monitored with PSMA PET/CT [10]. To illustrate this application, we present the following clinical case where ^18^F-Pylarify PSMA PET/CT guided restaging and management of a patient with known prostate cancer and brain metastases.

## Case presentation

**Fig. 1:**
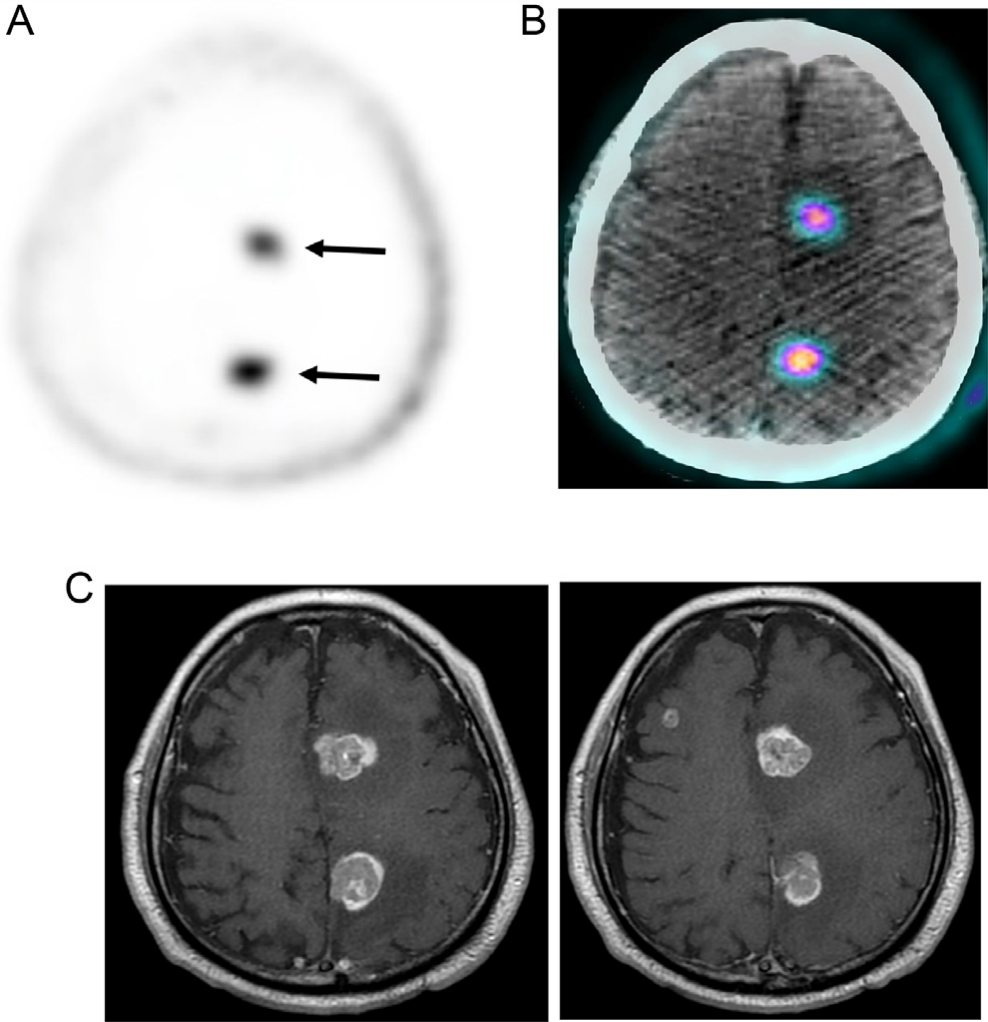
(A) Baseline axial PSMA PET brain lesions with SUV 5.0 and 7.8 and (B) PET/CT fusion image. (C) Brain MRI T1 with contrast shows enhancing lesions.

A 71-year-old man presented in 2020 with dysuria and an elevated PSA of 7.87 ng/mL. Prostate biopsy revealed prostate adenocarcinoma, Gleason score 9, with small cell and ductal features. Staging scans demonstrated metastases to lungs, consistent with stage IVB disease (cT3b, cN1, cM1). He underwent treatment with androgen deprivation therapy, taxanes, immunotherapy, and chemotherapy. His PSA had a good response, decreasing to undetectable levels. However, it recently rose to 0.68 and 0.53 ng/mL over the past 3 months. Restaging PSMA PET/CT on February 2, 2022 demonstrated retroperitoneal lymphadenopathy and osseous metastases, but the brain was normal. In May 2023, a repeat PSMA PET/CT revealed two large new brain lesions in the left parasagittal region (Figs. 1A and B), with standard uptake value (SUV) 5.0 and 7.9. He remained neurologically asymptomatic. Given the rapid development of these brain lesions, they were deemed metastatic. MRI of the brain obtained shortly after (Fig. 1C) showed 2 enhancing left hemispheric parasagittal lesions (2.5 and 2.2 cm) with surrounding vasogenic edema, along with 3 smaller lesions.

**Fig. 2:**
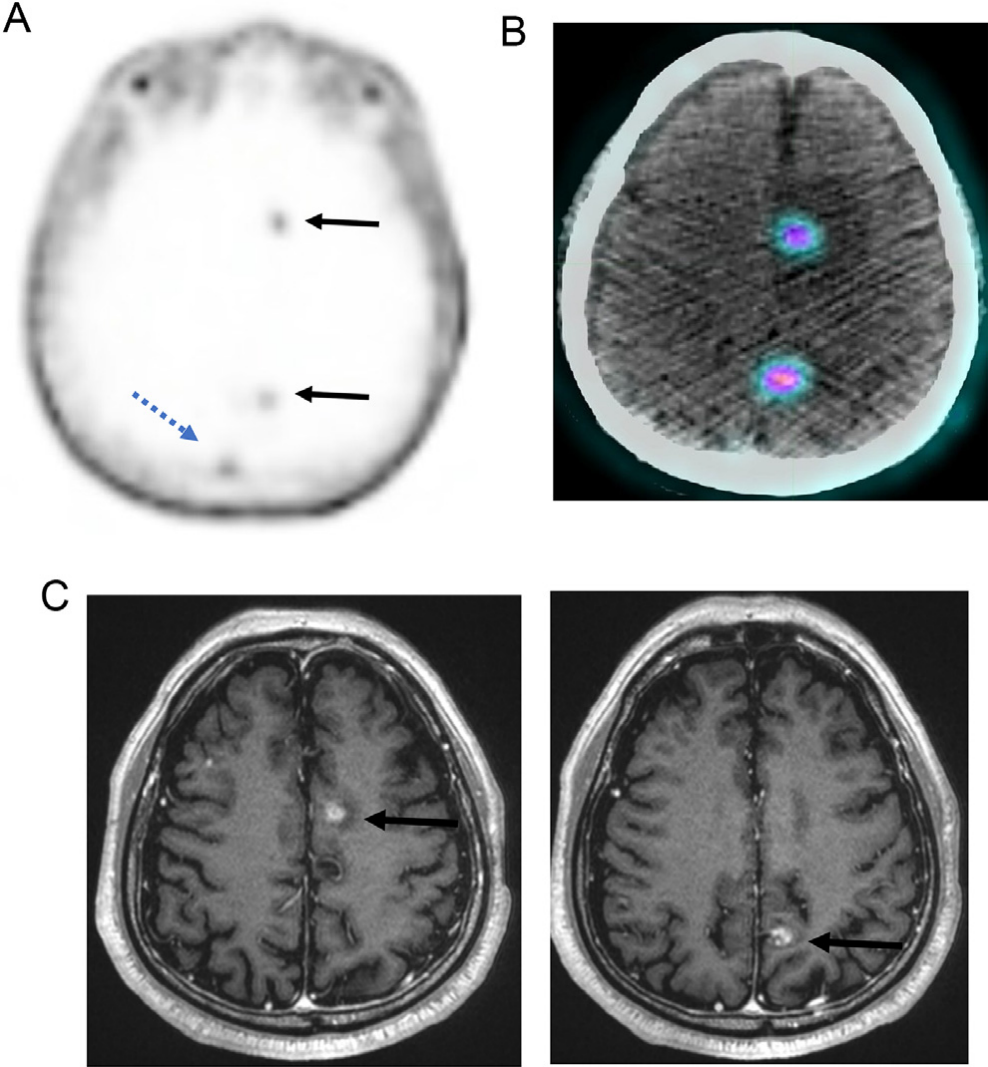
(A) PSMA PET post radiation shows regression of lesions with SUV 1.2 to 1.4 (solid arrows) similar to blood pool SUV 1.4 (dotted arrow) over venous sagittal sinus. (B) PET/CT fusion image shows lesions with low activity compared to baseline. (C) MR T1 with contrast post radiation shows dramatic reduction in lesion size.

The patient was referred for radiation therapy. He underwent whole-brain radiation receiving a total dose of 3000 cGy over 10 fractions. Follow-up PSMA PET/CT after radiation demonstrated significant reduction in size and activity of the irradiated brain lesions, with SUV values lower than blood pool (1.4 and 1.5), suggesting loss of PSMA-avid tumor (Fig. 2A). An MRI of the brain with contrast showed dramatic response, with only 9- and 8-mm lesions remaining (Fig. 2B) and decreased surrounding edema.

Clinically, at completion of brain radiation, the patient had mild right arm and leg weakness with stable gait that eventually resolved. His osseous metastases were no longer PSMA-avid on PET/CT. However, he still had residual PSMA-avid retroperitoneal adenopathy and soft tissue lesions not shown here. In summary, this case demonstrates the use of PSMA PET/CT for detecting PSMA-avid brain metastases that responded favorably to radiation therapy.

## Discussion

Once prostate cancer is diagnosed, prostate MRI and radionuclide bone scan are conventional used for staging newly diagnosed intermediate and high-risk disease to evaluate extra-prostatic extension and metastatic disease [11]. However, for lymph nodes and distant metastases, PSMA PET/CT has emerged as a superior radionuclide imaging technique compared to conventional imaging. PSMA-targeted radiotracers enable sensitive detection of primary tumors, metastases, and recurrent lesions even in those with low PSA levels and small tumor volumes [12].

Distinguishing brain tumor recurrence or progression from radiation-induced inflammatory and necrotic changes poses a major diagnostic challenge in conventional neuro-oncologic imaging, regardless of modality [13].

The key advantage of PSMA PET is high tumor-to-background contrast in the brain due to absent or low PSMA expression on normal brain tissue [14]. However, Studies about the use of PSMA targeted PET/CT in the evaluation for brain metastasis treatment response in the irradiated brain are sparse.

This case demonstrates the emerging role of ^18^F-Pylarify PET/CT for diagnosis and follow up of prostate cancer brain metastases treated with radiotherapy. The PSMA-avidity on the PET/CT scan clearly indicated a brain metastasis. Furthermore, the low PSMA-avidity on the postradiotherapy PET/CT scan clearly showed successful treatment.

PSMA-targeted PET radiotracers provide superior diagnostic accuracy compared to previous generation choline-based tracers and Fluciclovine [15]. The data from current literature show similar, comparable results when using Ga68-based and F18-based PSMA PET radiotracers based on their clinical impact in metastatic prostate cancer [16]. Furthermore, no data is available to differentiate their role in brain metastases from prostate cancer. As these PSMA-directed agents share a common target, we presume similar clinical utility among them in patients with prostate cancer and brain metastases.

## Conclusion

Our case highlights the use of F18 DCPF PYL PSMA PET/CT to detect brain metastasis and evaluate for prompt response to radiotherapy. An invaluable application particularly in the previously irradiated brain where MRI can be equivocal in distinguishing post radiotherapy necrotic and inflammatory changes from progressed or recurrent brain metastasis.

## Patient consent

We confirm that informed consent for publication of this case report was obtained from the patient.
